# Healthcare-associated infections and conditions in the era of digital measurement

**DOI:** 10.1017/ice.2023.139

**Published:** 2024-01

**Authors:** David C. Classen, Chanu Rhee, Raymund B. Dantes, Andrea L. Benin

**Affiliations:** 1 Division of Epidemiology, University of Utah School of Medicine and IDEAS Center VA Salt Lake City Health System, Salt Lake City, UT, USA; 2 Department of Population Medicine, Harvard Medical School and Harvard Pilgrim Health Care Institute, Boston, MA, USA; 3 Division of Infectious Diseases at Brigham and Women’s Hospital, Boston, MA, USA; 4 Division of Hospital Medicine at the Emory University School of Medicine, Atlanta, GA, USA; 5 Division of Healthcare Quality Promotion at the Centers for Disease Control, Atlanta, GA, USA

## Abstract

As the third edition of the *Compendium of Strategies to Prevent Healthcare-Associated Infections in Acute Care Hospitals* is released with the latest recommendations for the prevention and management of healthcare-associated infections (HAIs), a new approach to reporting HAIs is just beginning to unfold. This next generation of HAI reporting will be fully electronic and based largely on existing data in electronic health record (EHR) systems and other electronic data sources. It will be a significant change in how hospitals report HAIs and how the Centers for Disease Control and Prevention (CDC) and other agencies receive this information. This paper outlines what that future electronic reporting system will look like and how it will impact HAI reporting.

## History of HAI reporting

Surveillance and reporting of healthcare-associated infections (HAIs) in the United States have evolved substantially over the past half-century. Although some hospitals began to establish infection control programs with active surveillance for HAIs in the 1960s, a national reporting system was absent until 1970 when the Centers for Disease Control and Prevention (CDC) established the National Nosocomial Infections Surveillance System (the precursor to the current National Healthcare Safety Network [NHSN]).^1^ This system initially had just 62 participating hospitals but expanded rapidly in the ensuing years as HAI surveillance continued to gain momentum, propelled by the addition of infection surveillance and control programs to The Joint Commission’s hospital accreditation requirements in 1976 as well as by evidence that such programs were associated with lower facility-level HAI rates.^2,3^

The release of the Institute of Medicine’s “To Err is Human” report in 1999 documenting that HAIs were the leading cause of preventable harms in hospitals spurred even greater attention and catalyzed legislative mandates for public HAI reporting in several states beginning in 2002.^4^ The Deficit Reduction Act of 2005 resulted in a series of Centers for Medicare & Medicaid Services (CMS) payment reforms codified in the 2008 Medicare Hospital-Acquired Conditions program and the subsequent 2012 Medicaid Healthcare-Acquired Conditions program. These programs ceased hospital payments for care resulting from preventable conditions acquired in the hospital (including several HAIs) as identified by the *International Classification of Diseases, Ninth Revision, Clinical Modification* (ICD-9-CM) codes.^5,6^

Administrative codes have long been used for quality-of-care metrics given their availability, ease of use, and low cost. However, administrative billing data are known to have low sensitivity and low positive predictive values for certain HAIs.^7–11^ Because billing staff determine ICD codes based on medical record documentation, variability in HAI reporting may also occur.^12,13^ Furthermore, changes in documentation and billing practices can easily affect apparent HAI rates, particularly when financial incentives are at stake. This issue is underscored by studies demonstrating sudden declines in billing rates for certain HAIs targeted by Medicare after implementation of the Hospital-Acquired Conditions program, without corresponding changes in infection rates as determined by standardized NHSN criteria.^14,15^

In 2010, CMS incorporated HAI prevention into the Hospital Value-Based Purchasing Program established under the Affordable Care Act and implemented in fiscal year 2013, such that HAI rates are now used to benchmark hospitals and inform pay-for-performance programs. Recognizing the limitations of administrative codes for HAI surveillance, CMS shifted to using NHSN definitions beginning with central-line–associated bloodstream infections (CLABSIs), followed by catheter-associated urinary tract infections (CAUTIs), *Clostridioides difficile* infections, methicillin-resistant *Staphylococcus aureus* (MRSA) bacteremia, and select surgical site infections (SSIs).^16^ Using NHSN to determine HAI rates improves accuracy, clinical relevance, and credibility compared to administrative data.

As the breadth and importance of HAI reporting to NHSN has evolved over time, so have the methods of surveillance. Traditionally, HAI surveillance has been performed by infection preventionists who conduct manual medical record reviews of all patients at risk for the specified HAI and apply standardized case definitions then manually enter data into the NHSN database. However, this process is highly resource intensive and risks underdetection of HAIs depending on the rigor and breadth of the case-finding approach.^17^ In addition, some HAI definitions are complex and leave room for subjective interpretation, confounding attempts to use them for hospital benchmarking.^18–24^

To overcome the drawbacks of traditional methods for surveillance for HAIs, particularly its resource intensiveness and imperfect interrater reliability, electronic surveillance methods have been increasingly explored and utilized. “Semiautomated surveillance” involves algorithms that select patients who are highly likely to have an HAI based on clinical data extractable from EHRs and subsequently requires manual confirmation; numerous studies have demonstrated how electronic screening based on microbiology results, antibiotic exposures, and discharge diagnosis codes (alone or in combination) can increase the efficiency—and potentially the interrater reliability—of HAI surveillance, including for CLABSIs, CAUTIs, and SSIs.^25–29^ Fully automated systems, in contrast, apply standardized definitions entirely using electronic data and eschew any manual medical record review. The potential advantages of allowing for fully standardized and efficient assessments of large population has led the CDC to increasingly explore this pathway, including a shift away from ventilator-associated pneumonia toward ventilator-associated events, and the recent development of a simple and automatable definition of hospital-onset bacteremia.^30,31^

## Evolution of digital quality measures

Measuring the quality of care is a critical component of assessing the overall value of care.^32^ Payors increasingly require that hospitals and ambulatory clinics measure and report on more and more quality measures.^33^ These quality measures can be used to identify opportunities to improve care and report performance, and they are increasingly tied by payors to reimbursement of healthcare personnel (HCP). Before the electronic health record (EHR) era, these quality measures were manually abstracted and reported, constituting a significant burden on hospitals and clinics. This burden increased as the number of quality measures required for reporting expanded exponentially. In a 2013 analysis, a major academic medical center was required to report >120 quality measures to regulators or payors, and the cost of measure collection and analysis consumed ∼1% of the net patient-service revenue.^34^ Preliminary results from a survey of leadership in 20 healthcare systems, ranging in size from 180 to 3,000 beds, revealed that measurement activities required the equivalent of 50–100 full-time employees at each of these systems, at estimated costs ranging from $3.5 to $12 million per year.^35^

Interest in the use of electronic means such as EHRs to collect and report these quality metrics has grown due to the potential to reduce the burden of collecting and reporting these measures. The Health Information Technology for Economic and Clinical Health (HITECH) Act, passed in 2009 as a part of the American Recovery and Reinvestment Act, specified general guidelines for the development and implementation of a “nationwide health information technology infrastructure.”^36^ Through the HITECH Act initiatives, the federal government has spent resources to promote widespread adoption of EHRs that were intended to improve the quality, safety, efficiency, coordination, and equity of healthcare in the United States.^37^ A key feature of this program was the use of clinical quality reporting measures that would be collected and reported using certified EHR systems.^38^ These quality reports use standardized quality measures, as envisioned in the HITECH Act known as electronic clinical quality measures (eCQMs), to report hospital performance to healthcare payors. An electronic clinical quality measure (eCQM) is a clinical quality measure that is specified in a standard electronic format and is designed to use structured, encoded data present in the EHR.^38^ As EHR adoption has risen dramatically over the past decade, many HCP now must generate and submit these eCQMs through their EHR or another certified technology.

The initial rollout of eCQMs was challenging. Many hospitals and clinics assumed a significant burden from collection and reporting of quality measures with eCQMs that was heavier than with prior manual collection. This occurred for many reasons, including poor and contradictory measure specifications; EHR vendor software that was unable to collect and report these eCQMs; inaccurate calculation of measures by vendor software; and lack of key measure documentation in the EHR system, often requiring clinicians to enter more documentation into their workflows in the EHR.^35^ In a 2015 study, the estimated cost of EHR quality reporting for general internists, family physicians, cardiologists, and orthopedists was $15.4 billion annually.^39^ The accuracy of EHR-based quality reporting has also been shown to have mixed performance compared with manual chart review.^37^ Electronic quality reporting has been frequently inaccurate due to challenges in data completeness, accuracy, terminology use, gaps between structured fields and available free text, and inconsistency of measure logic implementation and EHR certification.^40^

In 2015, as these challenges were coming to light, the Institute of Medicine (IOM) released its report, *Vital Signs*, on national quality measurement and reporting that called for a significant reduction in the volume of different quality metrics reported and recommended the movement to eCQMs for all quality reporting.^41^ CMS responded to this report and to numerous stakeholder concerns about CMS eCQM quality reporting program with the development of their new digital quality measurement strategy.^42,43^ As part of this new digital measures program, CMS is updating and improving its existing eCQMs through a new program called the Centers for Medicare & Medicaid Services Electronic Clinical Quality Measure (eCQM) Strategy Project. This project began by outreach to working partners to identify burdens and recommendations related to the current eCQM implementation and reporting program, and then developed a series of initiatives to address these burdens and recommendations from frontline users.^43^ Six themes emerged from the stakeholder feedback. *Better Alignment* addressed the perceived lack of alignment across CMS programs, other payors, and regulatory agencies. *Improved Value* addressed the burdens with current reporting on eCQMs that HCP believe may not be relevant, do not contribute to their quality initiatives, or do not accurately represent the care they provide to patients. *Future Development Processes* addressed the burdens related to the length of time and lack of clarity of the eCQM development process. Measure developers and health IT vendors recommended a collaborative eCQM development environment to improve transparency for future measure needs and access to testing data to streamline the eCQM development lifecycle. *Integrated Implementation* and *Reporting Processes* addressed the complex eCQM workflows resulting from: 1) difficulty interpreting the eCQM specifications and discerning which data elements must be captured in the clinician workflow; 2) documentation required for eCQM reporting that does not support patient care directly; and 3) multiple submission mechanisms and formats resulting in delays, poor submitter feedback, and lack of usability and consistency. Finally, an enhanced *EHR Certification Process* outlined that hospitals and clinicians expected the EHR certification process to ensure accurate and successful eCQM calculation, reporting, and submission to CMS. In total CMS and its partners addressed over 100 recommendations to improve the eCQM development, implementation, and reporting experience by creating multiple implementation strategies.^43^

These eCQM changes are part of the larger CMS plan to move all quality reporting to digital quality measures (dQMs). CMS's new digital quality framework is built around 4 key domains: advancing technology; enabling measure alignment; improving the quality of data, such as standardized data elements and validation programs; and optimizing data aggregation.^42,43^ The dQMs are an expansion of eCQMs that allows for data collected not only from EHRs but also from an array of other electronic sources. CMS defines dQMs as “quality measures, organized as self-contained measure specifications and code packages, that use one or more sources of health information that are captured and can be transmitted electronically via interoperable systems.” These data sources may include administrative systems, electronically submitted clinical assessment data, case management systems, EHRs, laboratory systems, prescription drug monitoring programs (PDMPs), instruments (eg, medical devices and wearable devices), patient portals or applications (eg, for collection of patient-generated data such as a home blood pressure monitor, or patient-reported health data), Health Information Exchange Organizations (HIEOs) or registries, and other sources.^42,43^

The increasing use of structured, Fast Health Interoperable Resources (FHIR)–formatted EHR data exchanged through FHIR application program interfaces (APIs) can be leveraged to greatly reduce longstanding challenges to quality measurement. Currently, implementing individual EHR-based measures requires HCP to install and to adapt measure calculation software in their respective EHR systems, which often use variable or proprietary data models and structures.^42,43^ This process is burdensome and costly, and it is difficult to reliably obtain high-quality data across EHR instances. FHIR is a recent set of health IT standards optimized for secure and rapid data interoperability based on widely used internet standards used across other industries. Once HCP map their EHR data (structured using a uniform FHIR standard) to a FHIR API (application program interfaces) to meet the 21st Century Cures Act requirements, it will be possible to exchange much of the foundational data needed for measures without significant additional HCP investment or effort.^43^ Learnings from these activities can be leveraged and applied to other digital data that live outside the clinical EHR, enhancing and expanding the use of data such as patient-reported outcomes (PRO) and patient-generated health data (PGHD) for quality measurement in the future.^42^ These advances in interoperability are also expected to enable the development of measure calculation tools (MCTs) for dQMs that use EHR data solely so HCP no longer will need to install measures one-by-one and update them annually in their unique EHR systems. MCTs can be self-contained tools executed by the HCP on-site and by multiple other key actors in measurement, including CMS, other payors, clinical registries, and data aggregators.^42^ In the future, improved interoperability of EHR and other digital health data can fuel a revolution in healthcare delivery and quality measurement that leverage healthcare data across all settings and data sources.

## Current NHSN HAI surveillance: Strengths and challenges

NHSN has grown into the primary and most widely used tracking system for HAIs in the United States, with enrollment increasing from 300 hospitals in 2006 to currently >38,000 healthcare facilities in all 50 states, the District of Columbia, and Puerto Rico. This surveillance for HAIs continues to remain a national priority,^44^ as HAIs still affect 1 of every 31 patients and lead to $28.4 billion in excess healthcare costs annually.^45,46^ Fundamentally, HAIs violate the central medical oath of “first, do no harm” and are a bedrock of national efforts to improve the safety of healthcare.

NHSN has been instrumental in driving the successful reduction in key HAIs over the past decade by supporting 3 pillars required for measurable reductions: actionable outcome data; evidence-based, concrete interventions (eg, standardized central-line insertion practices); and an empowered healthcare workforce to enact prevention strategies, including both bedside clinicians and leadership from infection preventionists and hospital epidemiologists to clinical leaders and facility administrators. The stress on this third pillar from the COVID-19 pandemic resulted in the rise of several key HAIs in the face of stress and over-extension of the healthcare workforce.^47^ The well-established NHSN surveillance network was also able to document and draw attention to the impact of the COVID-19 pandemic on patient safety and has been an essential part of reinvigorating efforts to improve patient safety outcomes in the pandemic era.^48^

The use of NHSN quality measures in accountability programs has been instrumental in providing incentive for healthcare facilities to drive improvements in HAIs. To successfully support these programs, NHSN relies on benchmarking healthcare facilities against each other using risk-adjusted, standardized infection ratios.^49^ To date, risk adjustment has relied on facility-level factors because of the practicalities of collecting data. Multiple authors have called for patient-level risk adjustment to benchmark facilities more fairly for accountability programs.^50^ However, providing risk-adjustment using patient-level factors requires collecting detailed patient-level data on all patients in the relevant cohorts—virtually all patients in healthcare facilities nationally. Prior to the current era of digital measures, such data collection was entirely impractical because requiring detailed, data collection for so many patients would be too burdensome. As we move into the era of electronic data collection and digital measurement, such an approach becomes imaginable, and patient-level risk adjustment becomes plausible.

The greatest challenge that NHSN has faced in growing and evolving surveillance and quality measurement has been the high burden that properly rigorous data-collection places on the healthcare facilities. Several of the established NHSN HAIs track device or procedure-related infections (CLABSI, CAUTI, and SSI) using detailed and comprehensive surveillance definitions that are designed to emulate a clinical diagnosis of infection. As a result, these surveillance definitions may contain patient signs or symptoms (eg, chills in CLABSI, costovertebral tenderness in CAUTI or purulent drainage in SSI), which are relevant for clinical diagnosis but are documented in nonstandardized ways and are challenging to locate in structured EHRs. Despite advances in EHRs over more than a decade, these HAIs require some level of manual adjudication, and the resulting reporting burden has been well documented.^51,52^ Furthermore, manual adjudication opens surveillance definitions to potential interrater variation and subjectivity.^53^

In the ever-advancing era of digital measurement, NHSN has committed to addressing these challenges by leveraging developments in data interoperability to improve and evolve surveillance for HAIs as well as offering rigorously defined and measured surveillance for patient-safety and quality outside the realm of HAIs.

## HAI reporting in the era of digital quality measures

Currently, NHSN has several quality measures that utilize the Clinical Data Architecture (CDA) framework for entirely electronic data transfer, including Antimicrobial Use/Antimicrobial Resistance, Late-Onset Sepsis and Meningitis, and the Multidrug-Resistant Organism (eg, Methicillin-resistant *S. aureus* [MRSA] bacteremia), and *C. difficile* Infection (ie, LabID modules). The CDA submission process, although reliable and well established, relies on facilities to package data collected by healthcare personnel into electronic submissions.

**Fig. 1.**
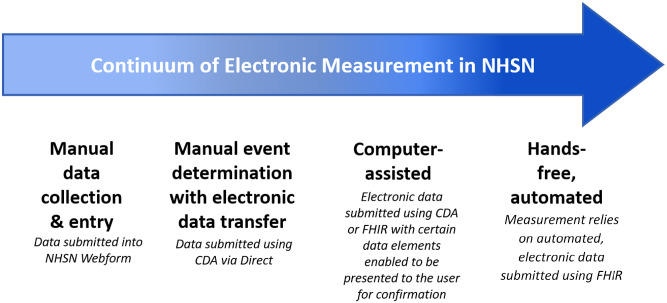
Continuum of electronic measurement in NHSN.

The next stage of NHSN electronic measurement will utilize both semiautomated and fully automated electronic surveillance for dQMs (Fig. 1). NHSN is developing a series of dQMs designed for data exchange via the FHIR. This approach will facilitate more robust, rapid, and efficient transfer of detailed healthcare data. Furthermore, because the approach enables the collection of patient-level data, NHSN will be able to evolve methods for risk adjustment while minimizing the burden of data collection. Importantly, NHSN will also be able to calculate balancing metrics that serve several functions. Balancing metrics can be used to identify whether the pressures created by measurement are causing unintended consequences, or they can monitor key process measures to evaluate the impact of key prevention practices. For example, alongside the planned measurement of healthcare-facility onset, antibiotic-treated *C. difficile* infection (HT-CDI), NHSN plans to track instances in which treatment for *C. difficile* infection was administered without a diagnostic test, which may indicate potential attempts to avoid recording HT-CDI events.

The first NHSN digital measures that leverage FHIR reporting are planned for release in 2023 and will address glycemic control, mirroring reporting that is currently specified as a CMS eCQM. Later in 2023, NHSN will add the ability to collect HT-CDI and hospital-onset bacteremia and fungemia (HOB) fully digitally. Additional HAIs, conditions, and patient-safety measures are under development. They address topics such as sepsis, respiratory infections, venous thromboembolism, and emergency preparedness.

## Opportunities and challenges as HAIs become digital quality measures

The progression of digital measurement presents new opportunities not only for patient-level risk adjustment and less burdensome data collection but also for more timely collection and reporting of surveillance metrics. The rapid data exchange enabled by tools like FHIR raises the question of how frequently HAI or other measure reporting in NHSN will take place, and how much closer NHSN can move to “real-time” reporting. Although the FHIR can enable nearly continuous data exchange, this cadence is likely not needed for most NHSN measures for several reasons. First, the creation of risk-adjusted quality measures may require inclusion of data elements such as administrative coding (which may also be used for excluding patients from a measure), which is typically not completed until after an encounter or admission is completed. Secondly, a higher frequency of data exchange with NHSN would need to be balanced against the computing burden with users’ FHIR servers. Facilities and users could utilize more frequent feedback of even preliminary events, such as daily new preliminary HOB events. However, on-site EHR tools or vender applications that leverage additional contextual local data may be better suited to provide near real-time feedback or clinical decision-making tools for improving the quality of care. For example, a local CDI prevention application could alert an infection preventionist to a daily list of preliminary HT-CDI events which could be analyzed to assess the physical distance of patients to each other, shared HCP, and records of specific cleaning practices, all of which go beyond what NHSN might need for HAI reporting.

Although FHIR is a transformative tool in data interoperability and may facilitate movement to dQMs with streamlined data exchange, it is important to acknowledge that advances in data interoperability cannot improve the quality of data documentation. For example, accurate electronic documentation of central lines is challenging.^23^ Until documentation practices improve, it will be challenging to bring these kinds of data elements into new dQMs and to transition legacy NHSN measures into dQMs. Standardized, comparable measurement of patient safety and quality will depend on the details of how each facility maps EHR data elements into FHIR resources so that the resulting data exchanged are consistent and standardized across all EHR implementations. Leveraging evolving FHIR standards and standardized terminologies will be essential to the success of FHIR-based dQMs.

NHSN is currently testing its FHIR gateway (called “NHSNLink”) with partner sites to validate and evaluate the process of data exchange. Efforts such as the US Core Data for Interoperability (USCDI) have been essential in prioritizing the first set of health data classes and elements for interoperable data exchange using FHIR and move the field toward realizing fully automated reporting. However, data submissions will require initial and ongoing validation to ensure that data are not missing or otherwise biased by any number of potential nuances or mappings of the EHR in each facility. NHSN is developing a validation structure, which will encompass both comprehensive validation prior to release of a measure as well as durable, ongoing validation as part of postimplementation routine activities.

Finally, there is a need to monitor how digital measurement leveraging new tools such as FHIR affects the engagement of frontline HCP. Current data abstraction by infection preventionists is labor intensive but often involves collaborations with frontline staff that are critical to driving improvement. Digital measurement, however, should not reduce the important need for education on how to use and interpret quality measures to improve care in individual units and facilities. NHSN program is committed to continuing to provide a comprehensive system for evaluating healthcare quality that includes data feedback and education.

In conclusion, advances in data interoperability have provided new opportunities for more robust digital quality measures while shifting the burden of reporting onto electronic systems and away from manual data collection. These new digital quality metrics include new HAI surveillance that will soon be incorporated into the CDC NHSN portfolio as well as measurement of patient safety beyond the realm of infection prevention. As the field continues to move forward, lessons learned from earlier efforts to digitize and automate quality measurement will be leveraged to achieve the vision of seamless, automated measurement that is also scientifically rigorous and valid and promotes the best possible care of patients.
